# Genome Stability: From DNA Repair Pathways to Integrated Genome Maintenance Networks

**DOI:** 10.3390/ijms27219448

**Published:** 2026-10-23

**Authors:** Tae-Hong Kang

**Affiliations:** Department of Biomedical Sciences, Dong-A University, Busan 49315, Republic of Korea; thkang@dau.ac.kr; Tel.: +82-51-200-7261

Genome stability is essential for the faithful transmission and expression of genetic information. Cells continuously encounter DNA lesions generated by endogenous metabolism, DNA replication and transcription, as well as environmental exposures. Although the molecular mechanisms of individual DNA repair pathways have been extensively characterized, recent studies increasingly emphasize that genome maintenance cannot be understood as a collection of independent repair reactions. Instead, genome stability emerges from the coordinated regulation of DNA replication, repair, transcription, chromatin organization, and cellular stress responses [1,2].

The articles published in this Special Issue, “Editorial Board Members’ Collection Series: Genome Stability”, reflect this expanding view of genome maintenance. Collectively, they address mechanisms ranging from lesion bypass and double-strand break repair to base and nucleotide excision repair, transcription-associated genome instability, chromatin regulation, environmental genotoxicity, and chromosomal abnormalities. Importantly, several contributions illustrate a broader trend in the field: the transition from pathway-centered models toward dynamic and interconnected mechanisms that determine how DNA lesions are processed in their physiological chromatin context.

One important theme is the close coordination between DNA replication, damage tolerance, and homologous recombination. Arbel-Groissman et al. (contribution 1) examined the role of proliferating cell nuclear antigen (PCNA) unloading in DNA damage bypass. Their study demonstrates that chromatin-bound PCNA itself can restrict homologous recombination and suggests that PCNA removal by the Elg1 RFC-like complex is a prerequisite for efficient recombinational repair. This work highlights how the physical residence and post-translational regulation of a replication factor can determine pathway choice following DNA damage.

The importance of pathway choice is also evident in the contribution focusing on the DNA helicase HELQ (contribution 2). HELQ possesses conserved helicase, DNA-binding, and strand-annealing activities and participates in multiple end-resection-dependent double-strand break repair pathways, including homologous recombination, single-strand annealing, microhomology-mediated end joining, synthesis-dependent strand annealing, and break-induced replication. Such multifunctionality illustrates an increasingly appreciated concept in genome stability research: repair proteins often do not belong exclusively to a single pathway but instead regulate multiple repair intermediates and thereby influence the balance between alternative repair outcomes.

A second major theme of this Special Issue is the evolving understanding of base excision repair (BER) as a context-dependent process rather than a constitutively uniform pathway. Pramanik et al. (contribution 3) investigated BER during mitosis and demonstrated that repair is relatively inefficient during this phase. They further showed that apurinic/apyrimidinic endonuclease 1 (APE1) remains associated with selected regions of condensed mitotic chromatin and contributes to efficient repair and rapid transcriptional reactivation following mitosis. These findings connect DNA repair with cell-cycle-dependent chromatin organization and suggest that genome maintenance proteins can serve functions extending beyond lesion removal to the restoration of transcriptional competence.

The clinical relevance of BER is further illustrated by Kladova and Kuznetsova (contribution 4), who examined human alkyladenine DNA glycosylase (AAG) and its relationship with cancer. Their analysis emphasizes that altered AAG activity and genetic variation may influence carcinogenesis, therapeutic resistance, and other disease phenotypes. Notably, their large-scale analysis of AAG variants identifies numerous potentially damaging polymorphisms and tumor-associated mutations, underscoring the growing importance of integrating biochemical DNA repair research with human genetic and cancer genomic data.

The boundaries traditionally assigned to individual repair pathways are also becoming increasingly blurred. Eom et al. (contribution 5) provide direct evidence for molecular communication between BER and nucleotide excision repair (NER) through an interaction between AAG and UV-damaged DNA-binding protein (UV-DDB). Their biochemical and structural analyses indicate that UV-DDB can interact directly with AAG and that DNA binding alters the configuration of this complex. The findings support a model in which UV-DDB may act as a broader damage-recognition or recruitment platform linking components traditionally assigned to separate repair systems. This study exemplifies a major current direction in genome stability research: repair pathways are increasingly being viewed as interconnected networks rather than rigidly separated biochemical modules.

A third prominent theme is the intimate relationship between transcription and genome maintenance. Jeon and Kang (contribution 6) review the emerging crosstalk between transcription-coupled repair (TCR) and R-loops. Although R-loops have important physiological functions in transcription and genome regulation, their persistent accumulation can interfere with transcription and replication and promote DNA breakage. The authors discuss evidence that TCR and R-loop processing pathways cooperate in maintaining transcriptional integrity and that disruption of this balance may contribute to cancer and neurodegenerative disease.

This concept is further extended in the review by Lee et al. (contribution 7), “Topology Resetting During Transcription-Coupled Nucleotide Excision Repair”. Rather than considering TCR solely as a sequential recruitment of repair factors to lesion-stalled RNA polymerase II (RNAPII), the authors emphasize the physical state of the transcription complex and its surrounding chromatin. They discuss a model in which Cockayne syndrome group B protein (CSB)-dependent remodeling, transcription-generated DNA supercoiling, topoisomerase activity, RNAPII ubiquitylation and clearance, and local chromatin architecture collectively influence the conversion of a stalled transcription complex into a repair-permissive substrate. This perspective reflects a broader shift in the field from describing the identities of repair factors toward understanding the mechanical, topological, and architectural constraints under which repair takes place.

Chromatin itself is increasingly recognized as an active component of the DNA damage response rather than merely a barrier to repair. Alekseev et al. (contribution 8) investigated the interaction of poly(ADP-ribose) polymerase 1 (PARP1) with nucleosomes using single-molecule optical tweezers and found that PARP1 binding can weaken interactions involving the inner DNA turn of the nucleosome even in the absence of PARP1 activation. These findings provide a physical basis for understanding how genome surveillance proteins can directly influence nucleosome stability and DNA accessibility. The increasing use of single-molecule approaches, structural biology, and high-resolution chromatin analysis is likely to reveal additional layers of regulation that cannot be captured by conventional pathway diagrams.

Genome instability is also strongly influenced by the cellular and environmental context. Zheglo et al. (contribution 9) examined chromosomal abnormalities arising in induced pluripotent stem cells (iPSCs) and identified recurrent chromosomal alterations and breakpoints associated with the *DCC* gene and *HIST2* histone gene cluster. Their results link these rearrangements to replication-stress-associated chromosome fragility and emphasize the importance of monitoring genome integrity during cellular reprogramming and expansion. This issue has practical implications for the safe application of stem-cell-derived models and regenerative medicine.

At the organismal level, Toprani et al. (contribution 10) address the potential consequences of cosmic ionizing radiation exposure in flight crews. Their review integrates epidemiological observations with current knowledge of DNA damage and repair and highlights important uncertainties regarding radiation dosimetry, mixed environmental exposures, and biological mechanisms underlying possible cancer risks in this occupational population. This contribution illustrates how fundamental genome stability research increasingly intersects with environmental health, occupational exposure, risk assessment, and public health policy.

Taken together, the studies in this Special Issue illustrate several emerging directions in genome stability research. First, the classical boundaries separating replication, repair, and transcription are becoming less distinct. PCNA unloading can control access to homologous recombination, UV-DDB may coordinate NER with BER, and transcription-associated R-loops can both participate in and threaten genome maintenance. Second, chromatin and DNA topology are moving from background variables to central mechanistic determinants of damage recognition and repair. Nucleosome dynamics, DNA supercoiling, chromatin accessibility, and higher-order genome organization are increasingly viewed as integral components of repair reactions. Third, genome maintenance must be considered in a cell-state-specific context. Mitosis, pluripotency, replication stress, transcriptional activity, and environmental exposure can profoundly alter both lesion generation and repair outcome.

Looking forward, several developments are likely to reshape the field. Advances in single-cell genomics, long-read sequencing, genome-wide damage mapping, proteomics, cryo-electron microscopy, high-resolution chromatin conformation analysis, and single-molecule biophysics will allow DNA damage and repair to be examined at unprecedented spatial and temporal resolution [3,4,5,6]. Integrating these technologies with functional genetics should clarify how repair pathway choice varies among genomic regions, cell states, and individuals. Equally important will be the integration of DNA repair biology with human genetic variation and cancer genomics, which may identify repair-factor variants that determine disease susceptibility or therapeutic response.

Another important challenge will be to understand genome stability as a systems-level property [7,8]. A lesion is not processed solely according to its chemical structure. Its fate is also determined by whether it is encountered by a replication fork or an elongating polymerase, its chromatin and topological environment, the availability of repair factors, cell-cycle stage, and the physiological state of the cell. Consequently, future models of genome maintenance will need to integrate lesion recognition, pathway crosstalk, chromatin remodeling, DNA topology, checkpoint signaling, and transcriptional recovery.

The contributions to this Special Issue therefore highlight a field that is moving beyond the concept of isolated DNA repair pathways toward an integrated view of genome maintenance networks. Understanding how these networks cooperate—and how their coordination fails in aging, cancer, inherited disorders, environmental exposure, and regenerative medicine—will remain a central challenge for genome stability research. We thank all authors and reviewers who contributed to this Special Issue and hope that this collection will stimulate further investigations into the molecular principles that preserve genome integrity.
